# Multicentric Retrospective Analysis of Oncocytic Adrenocortical Carcinoma: Insights into Clinical and Management Strategies

**DOI:** 10.1007/s12022-025-09857-0

**Published:** 2025-04-11

**Authors:** Antonio Prinzi, Valentina Guarnotta, Guido Di Dalmazi, Letizia Canu, Filippo Ceccato, Francesco Ferraù, Giuseppe Badalamenti, Manuela Albertelli, Maria Cristina De Martino, Giuseppe Fanciulli, Roberta Modica, Angelo Pani, Francesco Arcidiacono, Ignazio Barca, Francesca Donnarumma, Lorenzo Zanatta, Marianna Torchio, Ylenia Alessi, Chiara Vitiello, Francesco Frasca, Pasqualino Malandrino

**Affiliations:** 1 Endocrinology Unit, Department of Clinical and Experimental Medicine, Garibaldi-Nesima Medical Center, University of Catania, 95122 Catania, Italy; 2 Department of Health Promotion, Mother and Child Care, Internal Medicine and Medical Specialties, Section of Endocrinology, University of Palermo, Piazza Delle Cliniche 2, 90127 Palermo, Italy; 3 Division of Endocrinology and Diabetes Prevention and Care, IRCCS Azienda Ospedaliero-Universitaria Di Bologna, 40138 Bologna, Italy; 4 Department of Medical and Surgical Sciences (DIMEC), Alma Mater Studiorum University of Bologna, 40138 Bologna, Italy; 5 Department of Experimental and Clinical Biomedical Sciences, Endocrinology Unit, University of Florence, 50139 Florence, Italy; 6 Department of Medicine DIMED, University of Padova, Padova, Italy; 7 Endocrine Disease Unit, University-Hospital of Padova, Padova, Italy; 8 Department of Human Pathology of Adulthood and Childhood “G. Barresi” DETEV, University of Messina, Messina, Italy; 9 Department of Precision Medicine in Medical, Surgical and Critical Care (Me.Pre.C.C.), University of Palermo, Palermo, Italy; 10 Department of Internal Medicine and Medical Specialties (DiMI), University of Genova, Genova, Italy; 11 Endocrinology, IRCCS Ospedale Policlinico San Martino, Genova, Italy; 12 Dipartimento Di Medicina Clinica E Chirurgia, Università Federico II Di Napoli, Naples, Italy; 13 NET Unit, Department of Medicine, Surgery and Pharmacy, University Hospital of Sassari, Sassari, Italy; 14 Endocrinology, Diabetology and Andrology Unit, Department of Clinical Medicine and Surgery, Federico II University of Naples, 80131 Naples, Italy; 15 Endocrinology Unit, ASL Gallura, 07026 Olbia, Italy; 16 Department of Biomedical, Dental and Morphological and Functional Imaging Sciences, University of Messina, 98125 Messina, Italy

**Keywords:** Oncocytic adrenocortical carcinoma, Adrenocortical carcinoma, Cancer, Recurrent disease, Overall survival, Mitotane

## Abstract

Oncocytic adrenocortical carcinoma (OAC) is a rare variant of conventional adrenocortical carcinoma (ACC), characterized by oncocytic tumor cells comprising more than 90% of the tumor. Due to its rarity, there is a lack of reliable data on the clinicopathological features and outcomes of OAC. The aim of this study was to assess the clinical presentation, treatment modalities, and outcomes of patients with OAC, comparing these results with a cohort of patients with conventional ACC. Data from 9 referral centers in Italy on 44 patients with OAC were retrospectively analyzed and compared with data from 145 patients with conventional ACC. Patients with OAC had a smaller median tumor size, more favorable resection margin status, and lower incidences of venous invasion and persistent/recurrent disease during follow-up. Additionally, patients with OAC exhibited longer times to progression (TTP) and overall survival (OS) compared to patients with conventional ACC. Multivariable analyses identified Ki67 and tumor size as features independently associated with disease progression during post-surgical follow-up, while Ki67 and distant metastases at diagnosis were independently associated with OS in OAC patients. After complete tumor removal, the risk of recurrent disease was higher in patients with either Ki67 ≥ 20% or ENSAT stage III/IV. OAC appears to have a more indolent clinical course and better prognosis than conventional ACC. Similar to conventional ACC, Ki67 remains a significant prognostic marker for OAC and, along with ENSAT stage, serves as a reliable biomarker for identifying patients who may benefit from adjuvant mitotane therapy.

## Introduction

Adrenocortical carcinoma (ACC) is a rare endocrine cancer with an incidence of 0.5 to 2 cases per million individuals per year, characterized by a bimodal age distribution. The incidence is higher in childhood and during the fourth to fifth decades of life, and it predominantly affects females more than males (F:M = 1.5:1) [1–3]. Patients with ACC have a poor prognosis, with a 5-year survival rate of 16–47%, dropping to less than 20% in cases of metastatic disease [4–6]. In most cases, diagnosis is due to the presence of hormone hypersecretion (40–60%), although it is rarely associated with paraneoplastic syndromes (i.e., fever, hypoglycemia). However, an increasing number of patients are diagnosed due to the incidental discovery of adrenal masses, which occurs in 10–15% of cases [7, 8]. Hormonal excess is mainly caused by cortisol overproduction and, less frequently, by mineralocorticoids, androgens, or estrogens, with combined hormonal excess also being possible. Non-functioning ACCs are often diagnosed due to symptoms caused by the abdominal mass, which can include back pain, abdominal pain, nausea, vomiting, and, less frequently, fever and weight loss [9, 10]. Based on their cytomorphological features, ACCs are classified into different subtypes: conventional, oncocytic, myxoid, and sarcomatoid [11]. Oncocytic ACCs (OACs) are defined by the presence of more than 90% oncocytic tumor cells [12]. The classic Weiss criteria, which are commonly used to differentiate benign from malignant adrenal cortical neoplasms in adults, are not applicable to OACs. Instead, a modified version of the Weiss classification system, known as the Lin-Weiss-Bisceglia (LWB) criteria, has been developed to evaluate oncocytic adrenal cortical neoplasms [11, 12]. According to this system, a neoplasm is classified as OAC if it meets at least one major criterion (> 5 mitoses/50 high-power fields, atypical mitosis, or vascular invasion) [11–13]. Due to the rarity of OAC, data on its clinicopathological features at diagnosis and patient outcomes are limited and often contradictory. Previous studies, based on a small number of patients, suggest that OAC is a more indolent disease, with better survival rates and delayed recurrence compared to conventional ACC [13–16]. The primary aim of this multicenter study was to assess the clinical presentation, treatment modalities, and outcomes of patients with OAC. The secondary aim was to compare these results with a large cohort of patients with conventional ACC.

## Materials and Methods

### Study Population

In this retrospective study, we analyzed data from a series of patients with ACC diagnosed at nine tertiary referral centers in Italy. Patients were selected according to the following inclusion criteria: (1) diagnosis of OAC or conventional ACC confirmed by surgical intervention, (2) diagnosis made after 2004 (the year the LWB criteria were implemented), (3) availability of data on histopathological features (including Ki67), treatment, disease status during follow-up and at the last control, and survival. Additional mandatory data included gender, age at diagnosis, resection status (R0, complete resection; RX, unknown resection margins; R1, microscopically positive tumor margins; R2, gross residual disease), hormonal hypersecretion status, tumor-related symptoms, date of recurrence or disease progression after primary surgery, European Network for the Study of Adrenal Tumors (ENSAT) staging. A total of 189 consecutive patients were diagnosed with ACC between 2005 and 2023 and were included in this analysis: 44 patients were diagnosed with OAC, and 145 patients with conventional ACC.

The study was conducted in accordance to the Declaration of Helsinki and received approval from the Institutional Review Board and Ethical Committee Catania 2 in Sicily, Italy (approval no. 63/CEL).

Time to progression (TTP) was defined as the time (expressed in months) from curative-intent resection to the first evidence of local or distant progression of disease. Overall survival (OS) was defined as the time (expressed in months) from the date of diagnosis to the date of death. Patients who remained alive at the time of the last follow-up were censored at the date of their last known follow-up.

### Treatment and Follow-Up

According to current guidelines [7], the standard surgical treatment for ACC comprised “en bloc” resection of the adrenal tumor, with or without adjacent organs if invasion was suspected. The first follow-up visit was performed 3 months after surgery and then every 3–6 months for the following 3 years, including biochemical and cross-sectional imaging (computed tomography (CT), magnetic resonance imaging (MRI), and [Fluorine- 18]fluoro- 2-deoxy-d-glucose (FDG)-positron emission tomography (PET) evaluations.

Mitotane therapy was initiated with either adjuvant or therapeutic intent, aiming to maintain plasma levels between 14 and 20 mg/L. Persistent disease was defined as the presence of detectable residual cancer following initial treatment, while recurrent disease was defined as occurring later during follow-up. At follow-up, biochemical and/or structural disease was determined based on serum hormone levels and radiological findings.

Response to treatment was classified according to RECIST Criteria 1.1 [17], with categories including complete response (CR), partial response (PR), progressive disease (PD), and stable disease (SD).

### Pathological Examination

**Fig. 1:**
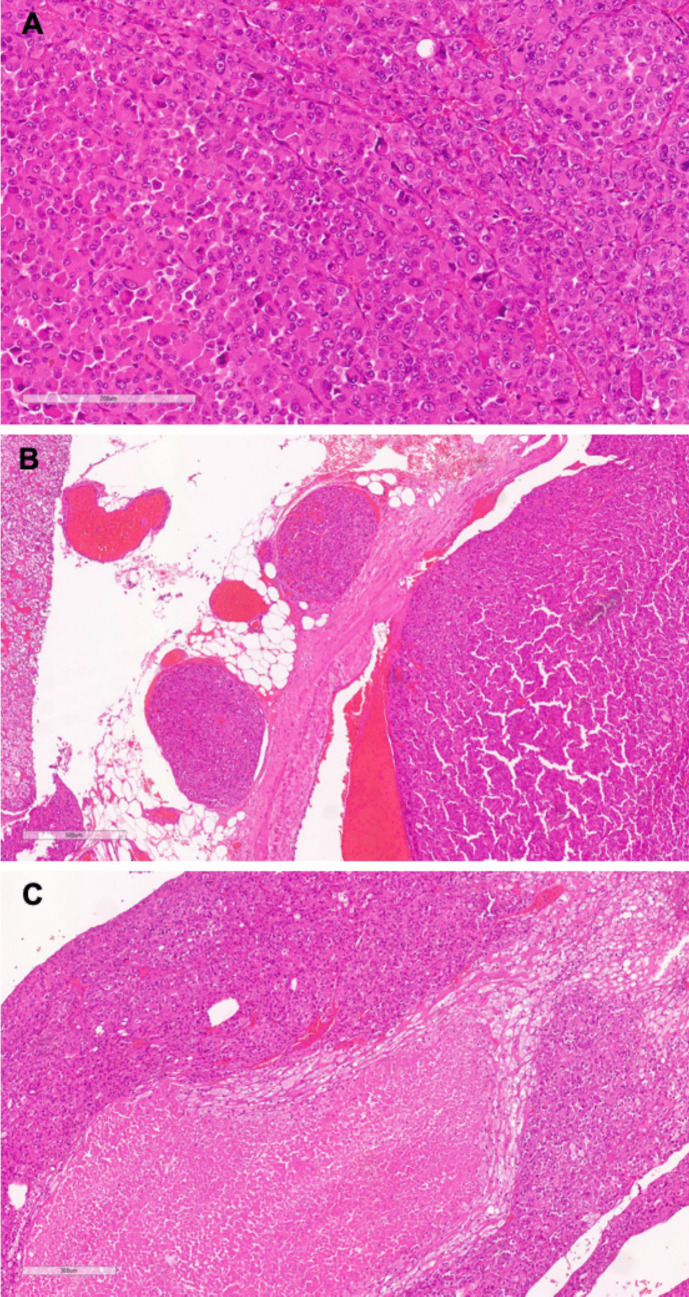
**A** The primary tumor of a patient with OAC. **B** Vascular invasion. **C** Necrosis

Histological samples were assessed by expert pathologists specializing in adrenal tumors. OAC was diagnosed when the adrenal neoplasms accounted > 90% of oncocytic tumor cells, and at least one major criterion of the Lin–Weiss–Bisceglia system was present: mitoses > 5 per 50 high-power fields (10 mm^2^), atypical mitoses, or venous invasion. The diagnosis of conventional ACC, in contrast, was based on the Weiss system, where the threshold for malignancy was a total score of ≥ 3 [18, 19]. The adrenal cortical origin of the tumors was verified using immunohistochemical markers (SF1, calretinin, synaptophysin, and Melan-A). Additionally, the Helsinki score was calculated as follows: 3 points for a mitotic count greater than 5 per 50 HPF, 5 points if necrosis was present, along with the absolute value of the Ki- 67 proliferation index. A Helsinki score > 8.5 was used for the diagnosis of malignant neoplasia. The mitotic count and Ki67 proliferative index were measured by manual counting in areas showing the highest proliferative activity. The mitotic count was assessed in the most mitotically active part of the tumor and recorded per 10 mm^2^, equivalent to 50 contiguous high-power fields. For the Ki67 proliferative index, 500–2000 tumor cells were counted, and the index was calculated by dividing the number of positive cells (any cell with nuclear staining was counted as positive) by the total number of malignant cells. The following features were recorded: tumor size, mitotic count, Ki67 index, resection status, and venous invasion. Figure 1 illustrates the primary tumor of a patient with OAC (panel A), along with vascular invasion (panel B), and necrosis (panel C).

### Statistical Analyses

Descriptive statistics were calculated for continuous variables as means ± standard deviations or medians with interquartile ranges (25 th–75 th percentiles) for non-normally distributed variables. Categorical variables were presented as frequencies and percentages. Comparisons between groups for continuous variables were performed using the *t*-test or the Mann–Whitney *U* test for variables that did not follow a normal distribution. The chi-square test was employed for categorical variables. To assess the relationship between disease outcomes and clinicopathological parameters, hazard ratios (HRs) with 95% confidence intervals (CIs) were estimated using both univariate and multivariable Cox proportional hazards analyses. Multivariable models were constructed by including all variables that were statistically significant in the univariate analysis. The final model was determined using a backward stepwise selection process. Kaplan–Meier survival curves were generated to estimate cumulative survival probabilities for the groups identified in the multivariable analysis. Survival time was defined as the time from diagnosis to either relapse, death, or the last follow-up for censored cases. The log-rank test was used to compare survival curves between groups. Cutoff values for potential predictor variables were determined using receiver operating characteristic (ROC) curve analysis. Statistical significance was defined as *p* < 0.05. All statistical analyses were performed using STATA version 18.0 (StataCorp LP, College Station, TX, USA).

## Results

### Clinical Features of Oncocytic Adrenocortical Carcinoma

Table 1 summarizes the clinicopathological features at diagnosis for the 44 patients affected by OAC included in this study. The mean age at diagnosis was 50.1 ± 15.3 years; 30 (68.2%) were female and 14 (31.8%) were male. The median tumor size at diagnosis was 6.7 cm (25–75 IQR = 5.0–10.0). Autonomous adrenal hormonal excess was observed in 22 patients (50.0%). Of these, 10 patients (22.7%) had mixed hormonal secretion (cortisol plus androgens in 9 patients and cortisol plus mineralocorticoid in 1 patient); 8 patients (18.2%) had autonomous cortisol production, and 4 patients (9.1%) had androgen excess. Of the remaining patients, 18 (40.9%) had non-functioning tumors, and in 4 cases (9.1%), this data was not available. The median Ki67 was 13% (25–75 IQR = 6–20), and the median mitotic count was 6/2 mm^2^ (25–75 IQR = 4–8). Moreover, we found that all patients with OAC except one had a Helsinki score > 8.5; the latter was considered malignant due to the presence of one major criteria (venous invasion) and three minor criteria of LWB (large size, capsular and sinusoidal invasion). After surgery, the majority of patients had R0 status (33 patients, 75.0%). RX status was identified in 7 patients (15.9%), and positive tumor margins (R1) were identified in 4 patients (9.1%). No patients had gross residual tumor (R2).

**Table 1:** Clinicopathological features at diagnosis in patients with oncocytic adrenocortical carcinoma and conventional adrenocortical carcinoma and comparison between groups

	OAC	Conventional ACC	*P* value
	*N* = 44	*N* = 145	
Sex, F/M (%)	30/14 (68.2/31.8)	98/47 (67.6/32.4)	0.94
Age at diagnosis, years (mean ± SD)	50.1 ± 15.3	49.5 ± 14.1	0.82
Tumor size (cm), median (25 th–75 th IQR)	6.7 (5.0–10.0)	9.3 (5.8–13.0)	0.044
Ki67, median (25 th–75 th IQR)	13 (6–20)	20 (10–30)	0.09
Mitotic count, median (25 th–75 th IQR)	6 (4–8)	6 (5–14)	0.95
Venous invasion, *n*. (%)	15 (34.1)	67 (46.3)	0.022
Necrosis, *n* (%)	33 (75.0)	122 (84.1)	0.17
Helsinki score, median (25 th–75 th IQR)	17 (10–28)	22 (13–35)	0.10
Hormonal excess, *n*. (%)			0.64
None	18 (40.9)	62 (42.8)	
Androgens	4 (9.1)	18 (12.4)	
Glucocorticoids	8 (18.2)	33 (22.8)	
Mineralocorticoids	0 (0)	2 (1.4)	
Mixed (gluocorticoid, androgens)	9 (20.4)	19 (13.1)	
Mixed (glucocorticoid, mineralocorticoids)	1 (2.3)	0	
Not available	4 (9.1)	11 (7.6)	
Distant metastases, *n*. (%)	6 (13.6)	35 (25.0)	0.11
Resection margin status, *n*. (%)			0.023
R0	33 (75.0)	75 (51.7)	
RX	7 (15.9)	42 (29.0)	
R1–2	4 (9.1)	28 (19.3)	
ENSAT stage, *n*. (%)			0.38
I	7 (16.3)	16 (11.9)	
II	17 (39.5)	51 (38.1)	
III	13 (30.2)	32 (23.9)	
IV	6 (14.0)	35 (26.1)	
Follow–up length (months), median (25 th–75 th IQR)	58.5 (32.2–81.5)	49.2 (22.5–90.6)	0.43
Persistence/recurrence during follow–up, *n*. (%)	15 (34.1)	75 (51.7)	0.040
Alive at the last follow–up, *n*. (%)	38 (86.4)	99 (69.2)	0.025

Regarding disease extension at diagnosis, only 6 patients (13.6%) presented with distant metastases: of these, three patients underwent surgery for hormonal control due to the secretion of cortisol and androgens, one patient for curative purposes due to infiltration of the ipsilateral kidney, and two patients to alleviate discomfort caused by the mass effect of the large tumor. According to the ENSAT staging system, 24 patients (55.8%) had localized disease (ENSAT I/II), 13 patients (30.2%) had locally advanced disease (ENSAT III), and 6 patients (14.0%) had metastatic disease at diagnosis (ENSAT IV).

Patients were followed up for a median time of 58.5 months (25–75 IQR = 32.2–81.5). After surgery, 30 patients (68.2%) were disease-free, 6 patients (13.6%) had persistent disease, and 9 patients (20.5%) experienced recurrence, with a median time to progression (TTP) of 31.4 months (25–75 IQR = 23.3–49.7). At the end of the follow-up period, 38 patients (86.4%) were alive. The survival rate at 5 and 10 years was 89.3% (95% CI = 73.3–95.9) and 76.5% (95% CI = 40.8–92.3), respectively.

### Comparison Between Oncocytic and Conventional Adrenocortical Carcinoma

The clinical and immunohistochemical characteristics of patients with OAC were compared with those of 145 patients with conventional ACC. Table 1 reports the comparison between the two groups.

At diagnosis, no significant differences were observed between the two groups in terms of male-to-female ratio, age, Ki67 proliferative index, mitotic count, Helsinki score, necrosis, hormonal excess, distant metastases, or stage. However, patients with OAC had a smaller median tumor size (6.7 cm vs. 9.3 cm, *p* = 0.044) and more favorable status of resection margins. Indeed, compared to conventional ACC, complete tumor resection (R0) was achieved in 75.0% of OAC patients versus 51.7% of conventional ACC patients, while micro- (R1) and macroscopic (R2) residual tumors were observed in 9.1% vs. 19.3%, respectively (*p* = 0.023). This difference remained statistically significant even when adjusted for tumor size.

Furthermore, patients with OAC had a lower incidence of venous invasion (44.1% vs. 66.3%, *p* = 0.022) and a lower incidence of persistent/recurrent disease during follow-up (34.1% vs. 51.7%, *p* = 0.040). As a result of these features indicating less aggressive biological behavior, patients with OAC had a higher likelihood of being alive at the end of the follow-up period (86.4% vs. 69.2%, OR = 0.36, 95% 95% CI = 0.14–0.90, *p* = 0.029).

**Fig. 2:**
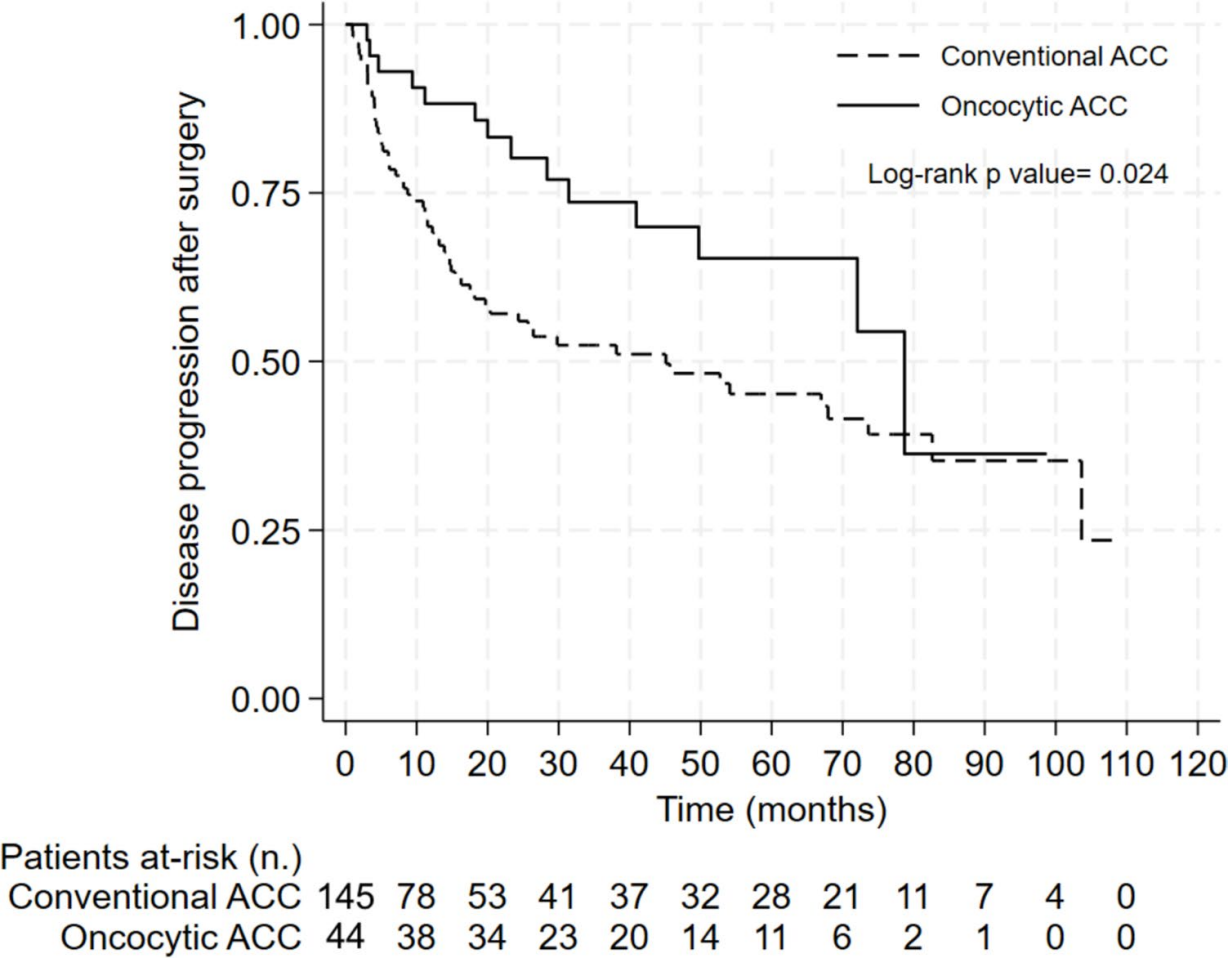
Kaplan–Meier curve for time to progression in patients with oncocytic and conventional adrenocortical carcinoma

**Fig. 3:**
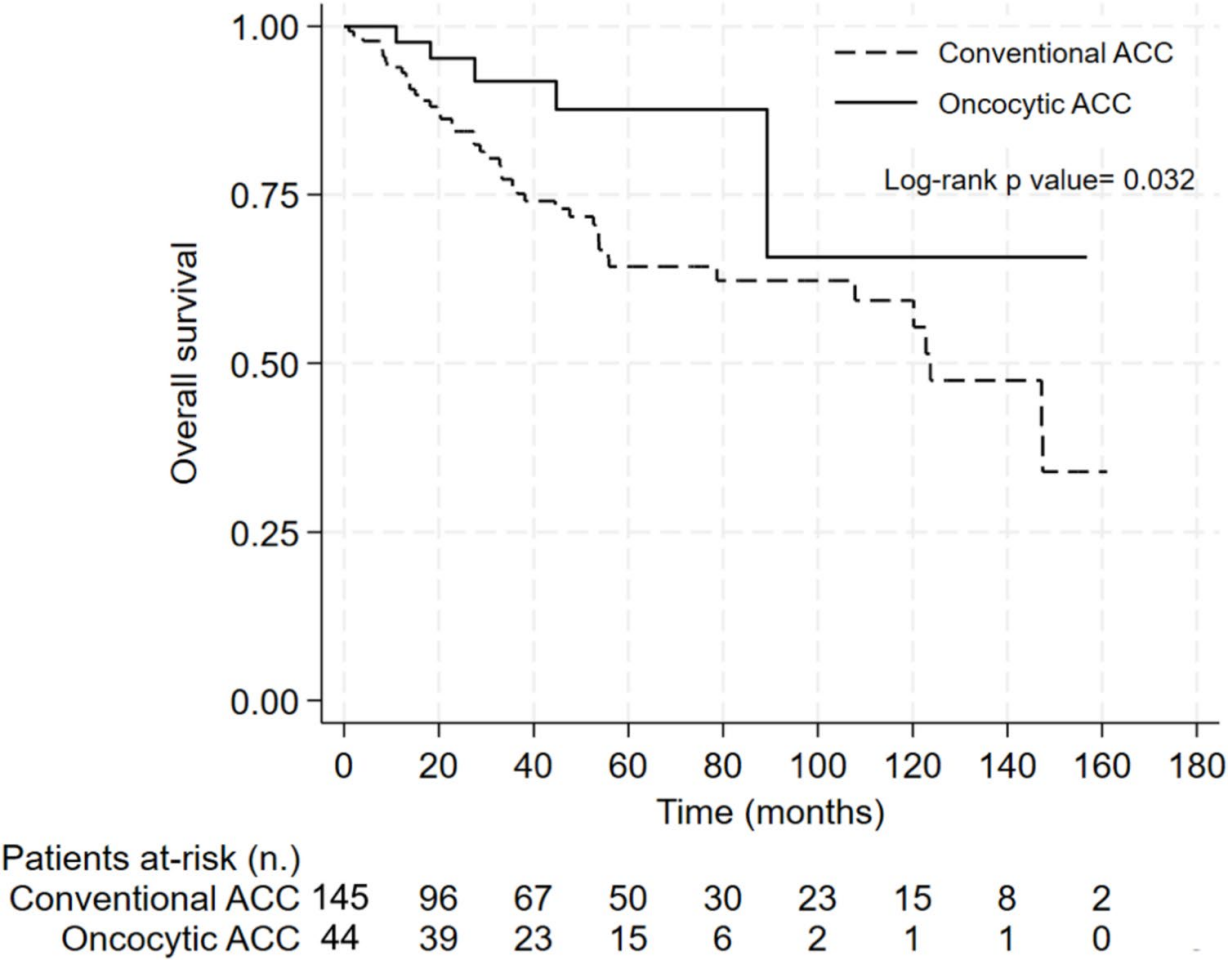
Kaplan–Meier curve for overall survival in patients with oncocytic and conventional adrenocortical carcinoma

Survival analysis demonstrated that patients with OAC had both a longer time to progression (TTP) and overall survival (OS) (78.7 months and not reached, respectively) compared to patients with conventional ACC (45.1 months and 123.7 months, respectively) (Figs. 2 and 3).

### Identification of Risk Factors for Persistent/Recurrent Disease and Overall Survival in Patients with OAC

Table 2 summarizes the characteristics associated with the risk of persistent/recurrent disease in patients with OAC. Univariate analysis revealed that progression was associated with the following factors: Ki67 (HR = 1.06, 95% CI = 1.02–1.10; *p* = 0.001), mitotic count (HR = 1.09, 95% CI = 1.03–1.17; *p* = 0.006), tumor size (HR = 1.14, 95% CI = 1.01–1.29; *p* = 0.041), distant metastases at diagnosis (HR = 6.62, 95% CI = 2.09–20.93; *p* = 0.001), stage III/IV ENSAT (HR = 8.32, 95% CI = 1.84–37.71; *p* = 0.006), and R1–R2 resection status (HR = 5.24, 95% CI = 1.08–25.47; *p* = 0.040). Multivariable analysis identified Ki67 (HR = 1.09, 95% CI = 1.04–1.15; *p* = 0.001) and tumor size (HR = 1.39, 95% CI = 1.08–1.77; *p* = 0.010) as features independently associated with disease progression during post-surgical follow-up. By ROC curve analysis, we found that a Ki67 cutoff value of 20% had the best performance in predicting the risk of progressive disease in patients with OAC (AUC = 0.73; sensitivity, 67%; specificity, 80%). Median TTP was 20.0 months in patients with Ki67 ≥ 20% and not reached in patients with ki67 < 20% (log-rank p value < 0.001).

**Table 2:** Cox regression analysis to evaluate factors associated with persistent/recurrent disease in patients with oncocytic adrenocortical carcinoma

	Univariate analysis			Multivariable analysis		
	Hazard ratio	95% CI	***P*** value	Hazard ratio	95% CI	***P*** value
Male sex	0.50	0.14–1.80	0.289			
Age at diagnosis	0.98	0.95–1.02	0.399			
Ki67	1.06	1.02–1.10	0.001	1.09	1.04–1.15	0.001
Mitotic count	1.09	1.03–1.17	0.006			
Tumour size	1.14	1.01–1.29	0.041	1.39	1.08–1.77	0.010
Necrosis	3.72	0.47–29.47	0.213			
Distant metastasis	6.62	2.09–20.9	0.001			
ENSAT stage III/IV	8.32	1.84–37.7	0.006			
R1 - 2 resection status	5.24	1.08–25.5	0.040			
Adrenal hormone excess	1.52	0.47–4.92	0.481			

Table 3 summarizes the characteristics associated with OS in patients with OAC. Univariate analysis identified the following factors: Ki67 (HR = 1.06, 95% CI = 1.01–1.12; *p* = 0.015), mitotic count (HR = 1.09, 95% CI = 1.01–1.19; *p* = 0.036), distant metastases at diagnosis (HR = 8.15, 95% CI = 1.28–51.92; *p* = 0.026), and R1–R2 resection status (HR = 13.04, 95% CI = 1.76–96.59; *p* = 0.012). Multivariable analysis confirmed that Ki67 (HR = 1.10, 95% CI = 1.01–1.19; *p* = 0.030) and distant metastases at diagnosis (HR = 23.84, 95% CI = 1.56–364.7; *p* = 0.023) were independently associated with OS.

**Table 3:** Cox regression analysis to evaluate factors associated with overall survival in patients with oncocytic adrenocortical carcinoma

	Univariate analysis			Multivariable analysis		
	Hazard ratio	95% CI	***P*** value	Hazard ratio	95% CI	***P*** value
Male sex	0.41	0.05–3.61	0.421			
Age at diagnosis	1.01	0.96–1.08	0.560			
Ki67	1.06	1.01–1.12	0.015	1.10	1.01–1.19	0.030
Mitotic count	1.09	1.01–1.19	0.036			
Tumour size	1.01	0.84–1.22	0.909			
Necrosis	0.78	0.07–8.95	0.837			
Distant metastasis	8.15	1.28–51.92	0.026	23.84	1.56–364.68	0.023
ENSAT stage III/IV	3.87	0.42–35.49	0.232			
R1 resection status	13.04	1.76–96.59	0.012			
Adrenal hormone excess	0.53	0.09–2.97	0.470			

### Identification of Factors for Adjuvant Mitotane in Patients with OAC

To identify factors that could help in selecting patients who might benefit from adjuvant mitotane, we analyzed 38 out of 44 (86.4%) patients who had complete tumor resection and negative post-surgical imaging. Among these 38 patients, 22 (57.9%) were treated with adjuvant mitotane, and 16 were not. Recurrent disease was identified in 10 (26.3%) patients, with a median TTP of 29.9 months (25–75 IQR = 20.0–49.7). Among these 10 patients, 7 were treated with adjuvant mitotane and 3 were not. The median Ki67 value was 15 (25–75 IQR = 8–23) in patients treated with adjuvant mitotane, compared to 9 (25–75 IQR = 6–10) in those not treated (*p* = 0.042). After disease progression on mitotane therapy: 4 patients underwent additional surgery, and at the last follow-up, 2 patients were alive and 2 had died after receiving systemic chemotherapy; 3 patients were treated with systemic chemotherapy, and at the last follow-up, 2 patients were alive and 1 had died; 3 patients were maintained on mitotane therapy because of a very slow tumor progression and were all alive at the last follow-up.

**Fig. 4:**
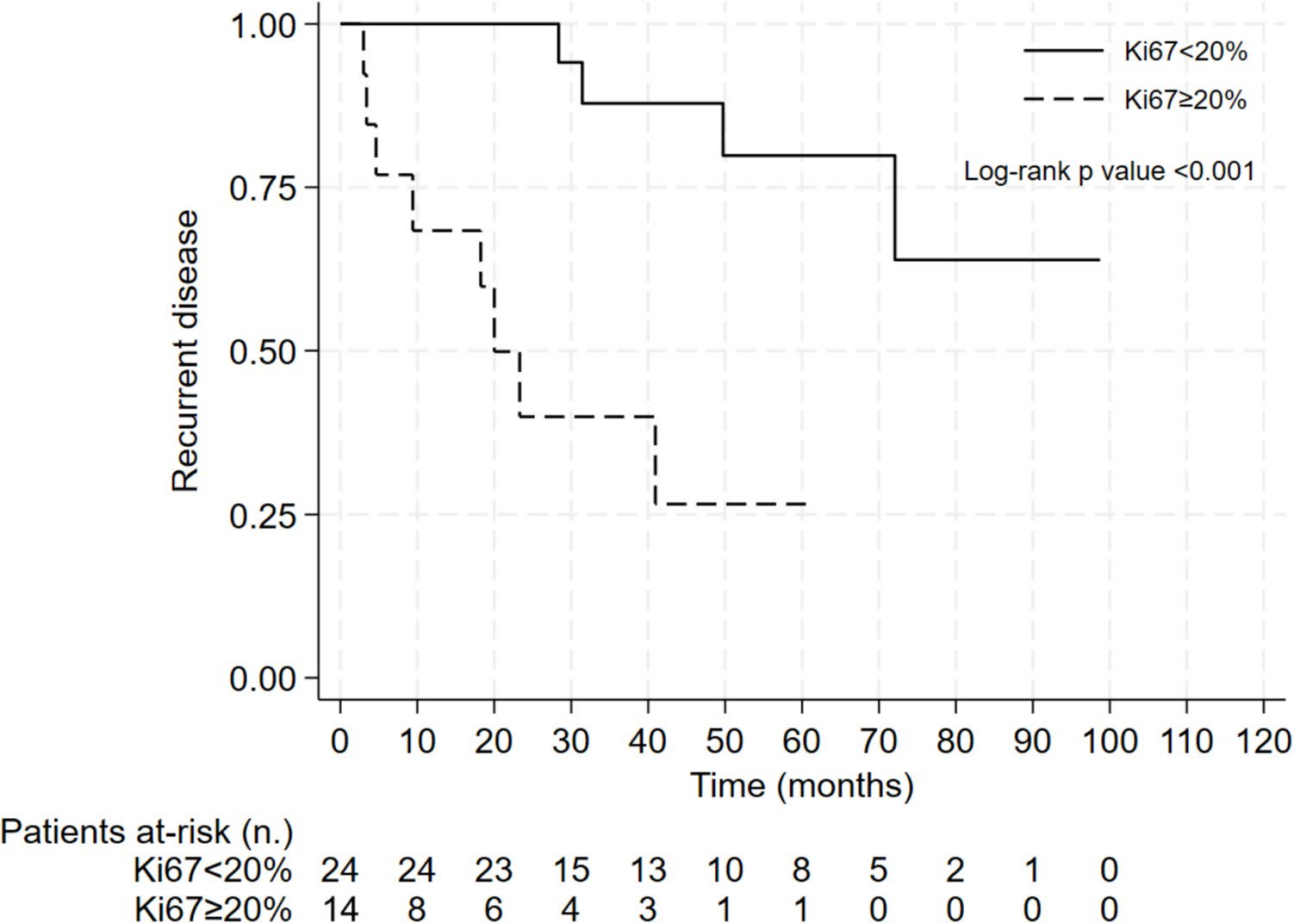
Kaplan–Meier curve for recurrent disease after surgery according to the Ki67 in patients with oncocytic adrenocortical carcinoma

**Fig. 5:**
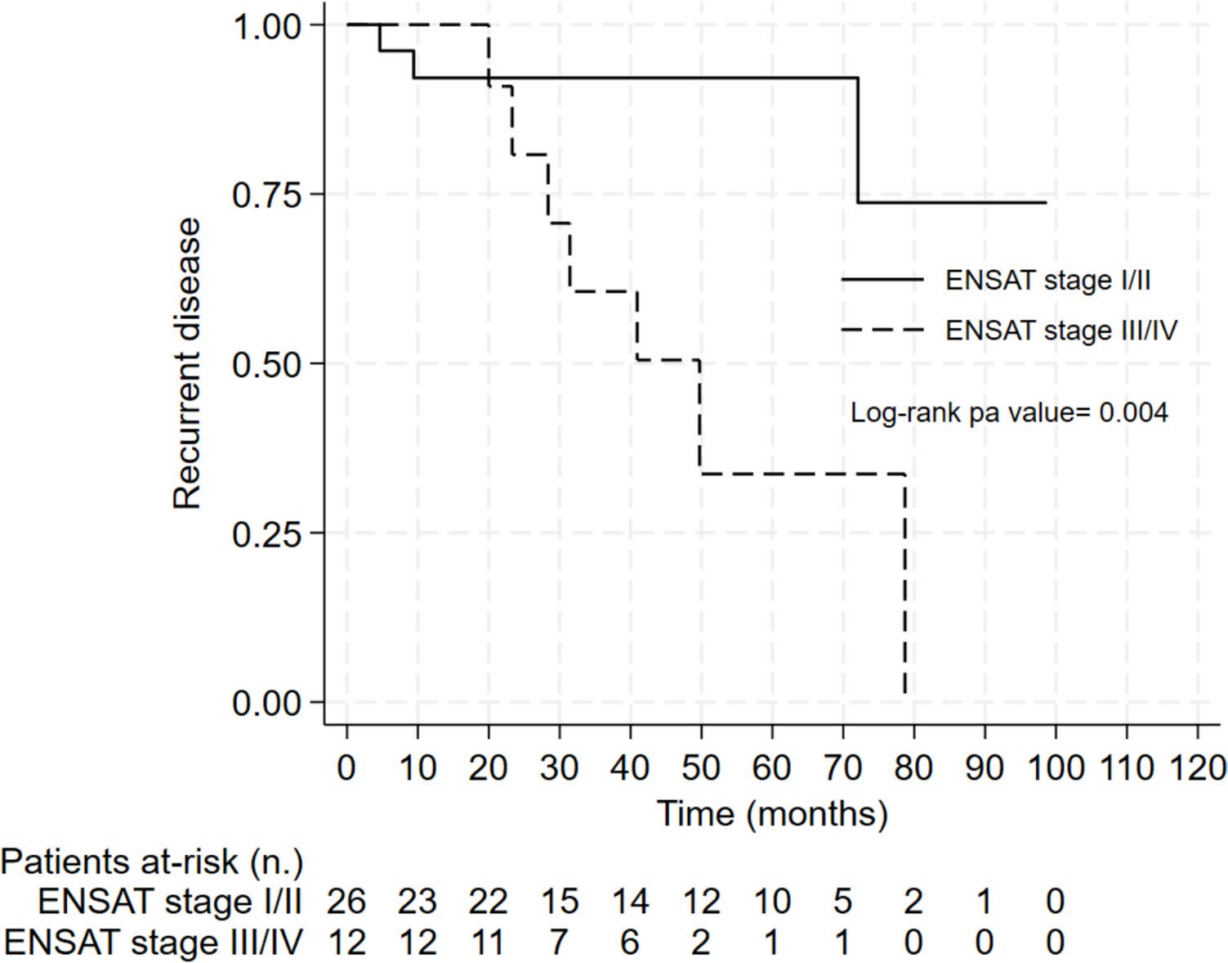
Kaplan–Meier curve for recurrent disease after surgery according to the ENSAT stage in patients with oncocytic adrenocortical carcinoma

At the univariate analysis, the risk of recurrence was associated with the following: Ki67 (HR = 1.06, 95% CI = 1.02–1.11; *p* = 0.006), mitotic count (HR = 1.15, 95% CI = 1.04–1.26; *p* = 0.006), and ENSAT stage III/IV (HR = 5.72, 95% CI = 1.45–22.49; *p* = 0.013). Multivariable analysis confirmed that Ki67 (HR = 1.10, 95% CI = 1.04–1.17; *p* = 0.001) and ENSAT stage III/IV (HR = 20.63, 95% CI = 2.37–179.37; *p* = 0.006) were independent factors associated with recurrent disease (Table 4). By ROC curve analysis, the Ki67 value of 20% was confirmed as the best in predicting the risk of recurrent disease in patients with OAC (AUC = 0.69; sensitivity, 56%; specificity, 83%). Figures 4 and 5 show the Kaplan–Meier curve for the probability of recurrent disease according to the Ki67 and ENSAT stage, respectively. Median TTP was 23.3 months in patients with Ki67 ≥ 20% and 49.7 months in patients with ENSAT stage III/IV (not reached in patients with Ki67 < 20% and ENSAT stage I/II; log-rank *p* < 0.001 and 0.004, respectively).

**Table 4:** Cox regression analysis to evaluate factors associated with recurrent disease in patients with oncocytic adrenocortical carcinoma

	Univariate analysis			Multivariable analysis		
	Hazard ratio	95% CI	***P*** value	Hazard ratio	95% CI	***P*** value
Male sex	0.85	0.22–3.31	0.817			
Age at diagnosis	1.00	0.96–1.06	0.844			
Ki67	1.06	1.02–1.11	0.006	1.10	1.04–1.17	0.001
Mitotic count	1.15	1.04–1.26	0.006			
Tumour size	1.12	0.96–1.31	0.151			
Necrosis	1.81	0.21–15.52	0.590			
ENSAT stage III/IV	5.72	1.45–22.49	0.013	20.63	2.37–179.37	0.006
Adrenal hormone excess	1.18	0.29–4.89	0.816

## Discussion

This multicentric study provides valuable insights into the clinical characteristics, treatment outcomes, and prognostic factors in patients with OAC. By comparing OAC with conventional ACC, our findings offer a deeper understanding of the differences in biological behavior, clinical progression, and survival rates between these two groups. OAC is a rare subtype of conventional ACC; however, when considering all adrenal oncocytic neoplasms, it may account for up to 30% [20]. It is worth noting that the overall incidence of OAC within the entire ACC population is currently unavailable. To our knowledge, this is the largest OAC cohort studied, making this data unique, as only a few studies in the literature have analyzed cohorts of patients affected by OAC.

The diagnosis of OAC poses a challenge for clinicians: it is unclear whether this variant confers a higher risk of aggressiveness, local or distant recurrence, or death compared to conventional ACC.

Existing knowledge comes from case series or small studies, where patients with OAC were often studied alongside those with benign or uncertain significance oncocytic adrenal neoplasms.

Previous studies [12, 13, 15, 16, 21] confirmed that OACs tend to present in middle-aged patients, with age at diagnosis similar to that found in this study, and females are slight predominant. They are mainly diagnosed as localized disease and have a higher rate of R0 margin status. On the other hand, hormonal secretion in OAC was detected in 50% of patients, with a significant portion of these exhibiting mixed hormonal excess, as noted in the study by Renaudin et al. [16]. This contrasts with some previous studies where OAC has been described as non-functioning in the majority of cases, suggesting that OAC may exhibit a more diverse clinical features than previously thought [13, 15, 21]. Interestingly, the median Ki67 reported in this study was similar to that reported in previous studies [15, 21].

One of the key findings of our study is the smaller tumor size at diagnosis and more favorable surgical outcomes in OAC compared to conventional ACC. The oncocytic variant was associated with a higher probability of complete tumor resection, as well as a lower risk of recurrent disease during follow-up and death, as indicated by our survival analysis. Despite the two groups showing similar values for Ki67, mitotic count, and tumor stage at diagnosis, patients with OAC had a 5-year survival rate of 87.9%, significantly higher than that of patients with conventional ACC. These data seem to confirm what some studies have previously suggested: the oncocytic variant exhibits less aggressive behavior than its conventional counterpart. Furthermore, the lower rates of venous invasion and fewer cases of positive surgical margins indicate the less invasive nature of OAC, contributing to better long-term outcomes [14, 15].

Exploring the subject further, we found that the risk of progressive disease was independently associated with Ki67 and tumor size. Although it is now well established that Ki67 is independently associated with recurrent disease in ACC and is the most important factor predicting recurrence in patients following R0 resection [22, 23], its role in OAC remains controversial. A higher Ki67 proliferative index appears to play a role in OAC: in the study by Ohtake et al. [24], the authors reported that a Ki67 ≥ 10% was correlated with the presence of significant histopathologic malignant characteristics, and in the study by Renaudin et al. [16], patients with poor outcomes had a median Ki67 of 14%. These findings emphasize the importance of these markers in guiding post-surgical management and follow-up strategies. In our series, we found that a cutoff of 20% best predicts the risk of persistent/recurrent disease.

Previous studies have reported that age ≥ 55, advanced disease stage, hormonally active ACC, RX/R1 status, Ki67 proliferative index, recurrence-free survival, and adjuvant mitotane treatment were independently associated with OS [25, 26]. Similarly, in our study of OAC, univariate analysis revealed that Ki67 proliferative index, mitotic count, distant metastases at diagnosis, and R1 status were related to OS; Ki67 and distant metastases were confirmed as significant in multivariable analysis. Therefore, our data confirms distant metastases as the strongest indicator of poor prognosis also in OAC, as for conventional ACC [7].

The diagnosis of OAC still remains a challenge: although the LWB criteria are the most widely used, the more recent Helsinki criteria and the reticulin algorithm have also found wide applicability over the years [11, 27, 28]. The study by Renaudin and colleagues [16] reported that the LWB criteria may overestimate the risk of malignancy for the oncocytic variant compared to Helsinki criteria: the latter were more accurate in identifying oncocytic adrenocortical tumors with a propensity for aggressive behavior. This high rate of malignancy diagnosis based on LWB criteria contrasts with findings from a meta-analysis in the literature, which validated this scoring system and reported a malignancy rate of 22% among 110 cases [13]. In the study Duregon et al. [28] involving 27 OAC, 40% of purely oncocytic tumors were classified as malignant using LWB criteria. However, this classification was not applied to mixed oncocytic adrenocortical tumors, which were instead assessed using the Weiss score. The higher malignancy rate observed in the study by Renaudin et al. with the Lin–Weiss–Bisceglia score may be attributed to the inclusion of both purely oncocytic and mixed oncocytic adrenocortical tumors (with an oncocyte component between 50 and 90%) [16]. Many researchers suggest that mixed tumors have a less favorable prognosis than purely oncocytic adrenocortical tumors, although there is no definitive data to confirm this assumption. Therefore, it remains still controversial whether LWB criteria overestimate the diagnosis of OAC; indeed, these findings could also imply that the LWB algorithm is less specific, potentially leading to the overclassification of tumors as malignant. However, in our study both the criteria showed similar accuracy to reliably diagnose this type of tumor as malignant. The reticulin algorithm has become increasingly favored in the evaluation of adrenal cortical neoplasms due to its consistent reproducibility [11]. This algorithm is becoming increasingly popular among diagnosticians due to its objective approach to assessing the tumor reticulin network and the minimal number of features required to determine malignancy [29]. Adrenocortical adenomas generally exhibit a well-preserved reticulin framework. However, when an altered reticulin network is observed in an adrenocortical tumor along with at least one additional criterion—such as vascular invasion, tumor necrosis, or a mitotic rate exceeding five per 50 high-power fields—the diagnosis of ACC can be established [30]. Also, for the oncocytic variant, the reticulin diagnostic algorithm appears to effectively identify clinically aggressive cases and help mitigate the risk of underestimating malignancy [28, 31].

Several biomarkers can be used for both diagnosis and prognosis in patients with ACC. An abnormal p53 and beta-catenin expression may be identified in a subset of ACC patients which are typically enriched in high-grade carcinomas that are reflected in poor-prognostic molecular clusters [32, 33]. *TP53* mutations are present in 20% of ACCs, and their detrimental effects disrupt the normal function of the p53 protein, impairing cell cycle regulation, DNA repair, and the control of cellular aging and apoptosis. Indeed, up to 80% of children with ACC are linked with Li-Fraumeni syndrome due to inactivating mutations of the tumor suppressor gene *TP53* [11, 34]. The insulin-like growth factor II (IGF2) system represents a crucial pathway in the tumor biology of ACC: even though the access to IGF2 immunohistochemistry is not widely available, his expression has been shown to be the best diagnostic ancillary tool in ACCs [11, 35, 36]. Due to the utility of these biomarkers, the newest WHO classification encourages pathologists to include, where available, these biomarkers for the diagnosis and prognosis of ACC.

The role of adjuvant mitotane therapy in OAC remains unclear due to the lack of large prospective trials or comprehensive retrospective series. Current guidelines for the management of ACC recommend adjuvant therapy with mitotane for patients with RX or R1 resection after surgery, as well as for patients with complete resection (R0) who are perceived to have a higher risk of recurrence, such as those with ENSAT stage III/IV or Ki67 > 10% [7, 37]. In our cohort, 57.9% of OAC patients received adjuvant mitotane. Although a direct survival benefit could not be conclusively demonstrated, we observed that the risk of recurrence was higher in patients with higher Ki67 values and advanced ENSAT stage (III/IV). These findings suggest that adjuvant therapy may be more beneficial for OAC patients with high-risk features, such as Ki67 ≥ 20% or ENSAT stage III/IV. Future studies are necessary to clarify the precise role of mitotane in OAC and to identify which patients are most likely to benefit from its use.

## Limitations

This study has several limitations. First, its retrospective nature may introduce selection bias, and the small sample size, particularly in the OAC group, limits the generalizability of our findings. As with any retrospective study, selection bias is inherent. For instance, by focusing exclusively on surgically resected cases, our findings may be biased toward low-stage disease, as adrenalectomy is typically reserved for patients with localized or oligo-metastatic disease. We identified 189 across nine referral centers from 2005 to 2023, which averages approximately 1.1 cases per year per center. A national cohort study spanning 12 Italian centers reported 512 ACC cases from 1990 to 2018, averaging about 1.4 cases per year per center [22]. Moreover, due to missing data in that cohort, only 310 cases were evaluated for the Ki67 index, corresponding to 0.9 cases per year per center. As outlined in our methods, our inclusion/exclusion criteria led to population size consistent with this benchmark.

Moreover, although we attempted to standardize the follow-up and treatment protocols across the participating centers, variations in clinical practice may have influenced the outcomes. Despite these limitations, our study represents one of the largest cohorts of OAC patients to date and provides important data that can inform future research and clinical practice. Future studies should aim to verify our findings in larger, prospective cohorts. Additionally, molecular and genetic studies of OAC could offer further insights into the unique biological characteristics of this subtype and potentially uncover novel therapeutic targets.

## Conclusion

In conclusion, our study demonstrates that OAC is a distinct subtype of ACC with a more indolent clinical course and better prognosis than conventional ACC. Complete surgical resection remains the cornerstone of treatment, and the Ki67 index, distant metastases at diagnosis, and tumor size are critical prognostic markers in OAC. Adjuvant mitotane therapy should be considered for patients with high-risk features; however, further research is needed to optimize treatment strategies and improve outcomes in this rare disease.

## Data Availability

No datasets were generated or analysed during the current study.
